# Chemo-free maintenance therapy in adult T-cell acute lymphoblastic leukemia: A case report and literature review

**DOI:** 10.3389/fphar.2023.1051305

**Published:** 2023-02-17

**Authors:** Yuanbin Song, Shuzhao Chen, Chenfei Liu, Lezong Chen, Weida Wang, Bingyi Wu, Yang Liang

**Affiliations:** Department of Hematologic Oncology, State Key Laboratory of Oncology in South China, Collaborative Innovation Center for Cancer Medicine, Sun Yat-sen University Cancer Center, Guangzhou, China

**Keywords:** chemo-free maintenance therapy, anti-programmed cell death protein 1, histone deacetylase inhibitor, T-cell acute lymphoblastic leukemia, case report, literature review

## Abstract

Maintenance therapy in adult T-cell acute lymphoblastic leukemia (T-ALL) is the longest phase but with limited option. The classic drugs used in the maintenance phase such as 6-mercaptopurine, methotrexate, corticosteroid and vincristine have potentially serious toxicities. Optimizing therapy in the modern age, chemo-free maintenance therapy regimens for patients with T-ALL may dramatically improve the maintenance therapeutic landscape. We report here the combination of Anti-programmed cell death protein 1 antibody and histone deacetylase inhibitor as chemo-free maintenance treatment in a T-ALL patient with literature review, thus providing a unique perspective in addition to valuable information which may inform novel therapeutic approaches.

## Introduction

T cell acute lymphoblastic leukemia (T-ALL) is an aggressive hematologic malignancy that accounts for about 25% of all new ALL cases in adults (Pui and Evans, 2006). With more effective pediatric-like multidrug line regimens being adapted to adults over the past decades, adult T-ALL has identified a subset of patients that benefits from said regimen and present with improved prognosis (Leoncin et al., 2022). The treatment for adult T-ALL including induction, consolidation, reinduction, delayed intensification and maintenance. Maintenance therapy in adult T-ALL is the longest phase but with limited options (Bassan et al., 2016). While the classic drugs used in the maintenance phase, daily 6-mercaptopurine (6-MP) and weekly low dose methotrexate (MTX) with or without monthly corticosteroid and vincristine pulses and periodic intrathecal injection (IT) chemotherapy, are required to decrease the risk of relapse, they also have potentially serious toxicities, including nephrotoxicity, life-threatening myelosuppression, gastrointestinal toxicity and hepatotoxicity (Rudin et al., 2017; Teachey et al., 2021). By optimizing therapy in the modern age, chemo-free maintenance therapy regimens for patients with T-ALL may dramatically change the maintenance therapeutic landscape.

## Case summary

A 26-year-old male was admitted to the local hospital with bilateral diffuse lymphadenopathy with the largest lymph node measuring 3 cm, without fever or family history of tumor. Routine blood examination revealed elevated white blood cell count of 21.5 × 10^9/L. Neck ultrasound revealed all cervical terminals had multiple, enlarged, bilateral, oval-shaped lymph nodes, with largest lymph node measuring 34 × 15 mm. The detailed admittance examination produced the following results: height- 171 cm, weight- 86 kg, blood pressure- 127/87 mmHg, pulse rate- 20/min and regular, temperature- 36.6.

**FIGURE 1:**
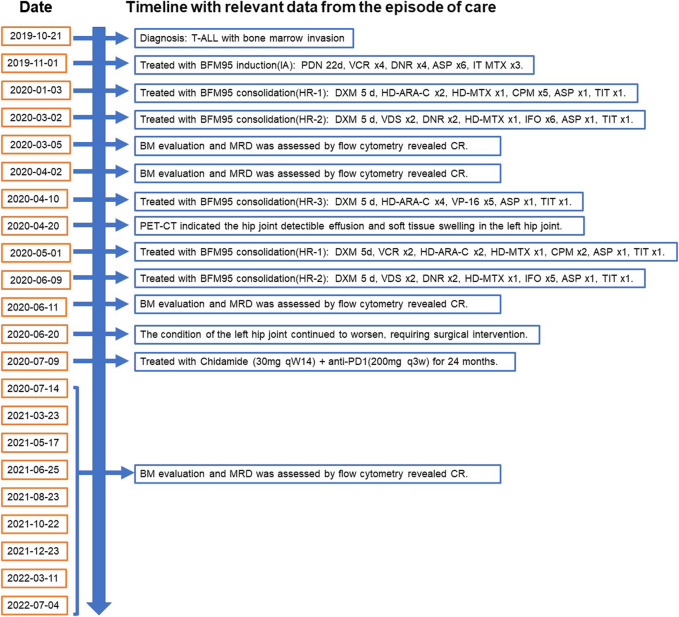
Timeline with relevant data from the episode of care, according to CARE case report guidelines. BM, bone marrow. MRD, minimal residual disease. BFM95, Berlin-Frankfurt-Münster (BFM)-ALL-95 regimen. CR, complete remission. PET-CT, Whole-body positron emission tomography scanning. HR, high risk; PDN, prednisone; VCR, vincristine; DNR, daunorubicin; ASP, *E coli* L-asparaginase; MTX, methotrexate; ARA-C, cytarabine; CPM, cyclophosphamide; VDS, vindesine; IFO, ifosfamide; DXM, dexamethasone; DOX, doxorubicin; HD, high dose; ID, intermediate dose; TIT, triple intrathecal therapy; VP-16, Etoposide.

Laboratory test results: WBC count- 21.5 cells/μL, platelet count- 245 cells/μL; Red blood cell count- 3.64 cells/μL. Whole-body positron emission tomography (PET) scanning showed that multiple enlarged lymph nodes in the neck, supraclavicular and infraclavicular areas, perihilar, mediastinum and groin. Immunohistochemistry stains of the cervical lymph node biopsy the cells were positive for LCA, CD5, TdT, CD10 and Bcl-2.21.99% of primary and immature cells were found in the bone marrow smear and express CD99, CD7, CD3, CD34, CD38, TdT, and CD5. According to National Comprehensive Cancer Network (NCCN) guidelines, the final diagnosis was T-ALL with bone marrow invasion belonged to the high-risk group. Thereafter, starting in November 2019, the patients underwent chemotherapy based on BFM95 protocol. In March 2020, the patient achieved complete remission (CR) by bone marrow smear and cerebrospinal fluid specimen from a lumbar puncture. However, in April of 2020, PET-CT scan showed the hip joint indicated ischemic necrosis of left femoral head, detectible effusion and soft tissue swelling in the left hip joint. Painful swelling of the left hip joint has rendered him unable to tolerate chemotherapy and long-term continuous treatment. Therefore, in order to effectively delay or halt the progression of the disease, it is necessary to try new and effective treatment-modifying alternatives. The patient was examined for programmed cell death protein 1 (PD-1) expression levels, as well as CD3^+^, CD3^+^CD4^+^, CD3^+^CD8^+^ T cell levels in peripheral blood (Table 1). We treated the patient with anti-programmed cell death protein 1 (PD-1) antibody Sintilimab every 3 weeks plus histone deacetylase inhibitor (HDACi) Chidamide twice a week as maintenance therapy for continually 24 months till now. The timeline with relevant data from the episode of care was demonstrated in Figure 1. The examination of minimal residual disease (MRD) showed that no abnormal phenotypic expression of primitive naive T lymphocytes was detected. Bone marrow (BM) evaluation revealed normal myelogram. Patient did not receive transplantation due to donor availability.

**TABLE 1:** PD-1 expression levels on lymphocyte.

	Expression	Normal range
CD3^+^	35	<25%
CD3^+^CD4^+^	9.9	1.9%–10%
CD3^+^CD8^+^	71.3	2.6%–42.7%

## Discussion

**FIGURE 2:**
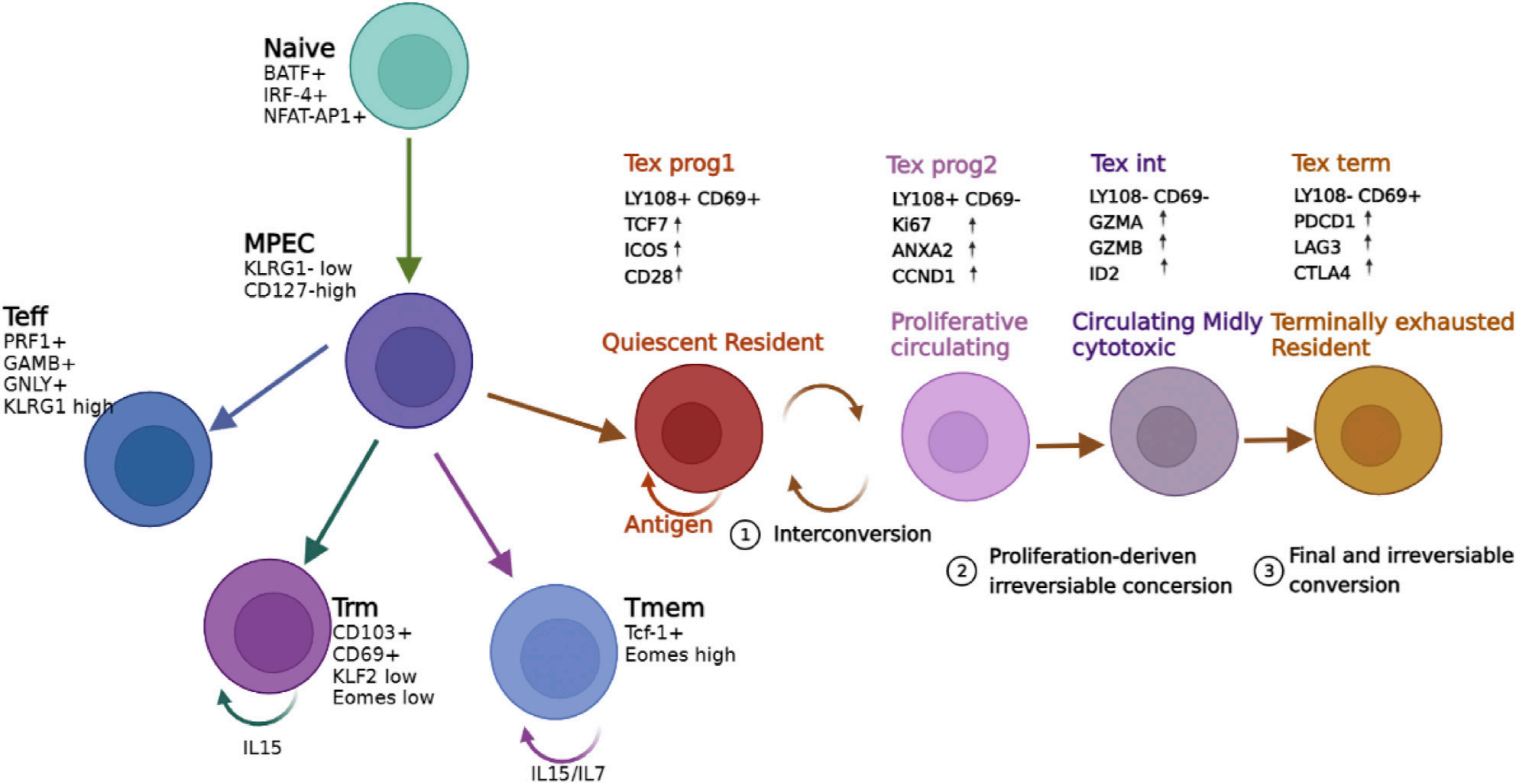
Schematic diagram of T cell activation and developmental relationships following antigen recognition. T eff, T effector cells; MPEC, memory precursor effector cells; TRM, tissue-resident memory T cells; Tmem, memory T cells; Tex Prog1:T cell exhaustion progenitors 1; Tex Prog2, T cell exhaustion progenitors 2; Tex Int, T cell exhaustion intermediate; Tex Term, T cell exhaustion terminally. The figure was adapted from McLane et al. (2015) and Beltra et al. (2020).

In the tumor microenvironment, T cell exhaustion is characterized by the progressive loss of effector function and reduced proliferative capacity triggered by persistent tumor antigen stimulation (Figure 2). During the initial activation of naive T cells by antigen presentation, several transcription factors including BATF, IRF-4, and NFAT-AP1, facilitate these activated cells differentiation toward KLRG-1 ^low^ CD127 ^high^ MPECs (memory precursor effector cells). MPECs have been previously shown to undergo multiple differentiation pathways, depending on antigen levels and disease environment. T-eff (effector) cells that form MPECs secrete many cytokines that directly exert effector functions with a high expression of high KLRG-1. TRM cells (tissue-resident memory T cells) display a memory phenotype, express characteristic cell-surface markers, such as CD103, CD69, but lacked expression of CD127. Tm (memory T cell) cells expressed Tcf1 and Eomes, which are transcription factors critical for the self-renewal ability (McLane et al., 2015). Persistent tumor antigen subverts CD8^+^ T cell differentiation toward exhaustion. Based on Ly108 (Slamf6) and CD69 expression, the developmental trajectory of CD8^+^ Tex cells can be divided into four stages (Figure 2), including quiescent resident stage (T cell exhaustion progenitors 1 (Tex Prog1), proliferative circulating stage (T cell exhaustion progenitors 2 (TexProg2), circulating mildly cytotoxic stage (T cell exhaustion intermediate (TexInt), and terminally exhausted resident stage (T cell exhaustion terminally (Tex Term) (Beltra et al., 2020). Tex Prog1 and Tex Prog2 are two interconverting TCF1+ progenitor cell, the former is quiescent and blood inaccessible, with high level of TCF, ICOS, and CD28, while the latter could initiate cell cycling and gained access to blood circulation, with high level of Ki67, ANXA2 and CCND1. Tex Prog2 gradually lost TCF1 and turn into TCF1− T-bethi intermediate Tex subset that regain the cytotoxicity, with a high level of GAMA and GZMB. These intermediate Tex cells terminally differentiate into Tex Term subset and exit the cell cycle and stop dividing, present with high level of immune checkpoint molecules, such as PDCD11, LAG3 and CTLA4. The TexProg2 and TexInt subsets were preferentially expanded in response to PD-1 pathway blockade and restore the cytotoxic function. Targeting the PD-1 pathway has shown to be efficacious treatment for T cell lymphomas (Kwong et al., 2017). There are increased clinical trials interrogating the efficacy of PD-L1 inhibitors (Table 2), both as single agents and in combination with existing treatments, in aggressive or relapsed/refractory T cell lymphoma (NCT03075553, NCT02631746 (Rauch et al. 2019), NCT03011814, NCT03161223 and NCT03046953, Table 2).

**TABLE 2:** Clinical trials of PD-1 in T cell lymphoma and HDACi in ALL.

Study ID	Years	Phase	Study design	Drug	Number of patients	Median age	Male/Female	Outcome	Center
NCT03075553	2017	II	Single-Arm	Nivolumab	12	65 (35–75)	6/6	Terminated	Mayo Clinic
NCT02631746 Rauch et al. (2019)	2015	II	Single-Arm	Nivolumab	3	51	2/1	Completed	Duke Cancer Institute LAO
NCT03011814	2017	I/II	Parallel Assignment	Durvalumab				Recruiting	City of Hope Medical Center
NCT03161223	2017	I/II	Randomized	Durvalumab				Recruiting	University of Virginia
NCT03046953	2017	II	Single-Arm	Avelumab	34			Completed	University of Birmingham
NCT00882206 Burke et al. (2014)	2009	A Therapeutic Trial	Single-Arm	Vorinostat	13	16 (3–54)		Terminated	Masonic Cancer Center
NCT01483690 Burke et al. (2020)	2011	A Pilot Study	Single-Arm	vorinostat	23	12 (1.6–21.4)	6/17	Terminated	Therapeutic Advances in Childhood Leukemia Consortium
NCT00816283	2009	I	Single-Arm	vorinostat	5			Completed	City of Hope Medical Center
NCT01383447	2011	I/II	Single-Arm	Entinostat	2		1/1	Terminated	National Cancer Institute (NCI)
NCT01312818	2011	A Therapeutic Trial	Single-Arm	Vorinostat	2		0/2	Terminated	Masonic Cancer Center
NCT00723203	2008	II	Single-Arm	Panobinostat	16	57 (18–81)	7/9	Terminated	City of Hope Medical Center

Blocking the PD-1/PD-L1 axis with anti-PD-1 antibody have been as a therapeutic strategy for T-cell lymphomas to avert effector T-cell dysfunction. For example, seven male patients with refractory NK/T-cell lymphoma failing l-asparaginase were treated with pembrolizumab and the ORR reached 100% (Kwong et al., 2017). A retrospective study revealed that ORR of 57% were achieved by pembrolizumab treatment in patients who previously received ≥2 chemotherapy regimens suffering from non-Hodgkin lymphoma (Li et al., 2018). In addition, an ORIENT-4 study reported that the ORR for R/R NKT patients based on Sintilimab-treatment was 68% (Tao et al., 2019). Currently, the PD-1 inhibitors pembrolizumab and nivolumab have been recommended for the treatment of R/R NKT by the NCCN guideline.

Histone deacetylation plays a key role in the epigenetic regulation of gene expression (Sandoval and Esteller, 2012). These discoveries have prompted the development of HDACi-based therapies designed to selectively target deregulated signaling pathways. At present, only six clinical studies have investigated the use of HDACi for ALL (NCT00882206 (Burke et al., 2014), NCT01483690 (Burke et al., 2020), NCT00816283, NCT01383447, NCT01312818, and NCT00723203, Table 2) and only one study has demonstrated that decitabine and vorinostat in combination with chemotherapy was tolerable and demonstrated clinical benefit in relapsed patients with relapsed/refractory acute lymphoblastic leukemia (Burke et al., 2014). Epigenetic mechanisms including changes in histone modifications are well-described as modulators of immune cell differentiation and function (Chen X et al., 2020).

Several studies have indicated that HDACi is a potential therapy against T-cell acute lymphoblastic leukemia. Previous basic studies provided compelling evidence that HDAC are involved in tumor development and progression and inhibitors of HDACs have potential anticancer activities in T-ALL. HDACi were shown to induce apoptosis in ALL cell lines *in vitro* (dos Santos et al., 2009). High expression levels of HDAC3, HDAC7 and HDAC9 are associated with poor prognosis in T-ALL disease (Moreno et al., 2010). HDACi can promote response of lymphoid malignancies to glucocorticoids (GCs) by reversing epigenetic silencing of BIM (Bachmann et al., 2010). Panobinostat, an HDACi belonging to the hydroxamate class, can induce apoptosis of ALL cells and distinctly prolong survival of xenogeneic mice models of human acute lymphoblastic leukemia (Scuto et al., 2008; Vilas-Zornoza et al., 2012). Moreover, panobinostat can effectively induce leukemic cell death in t (4; 11)-positive primary infant ALL cells through the inhibition of MLL-AF4 fusion product (Stumpel et al., 2012). However, some studies suggest that the HDACi may not work well in treatment of T-ALL. For example, the major epigenetic regulator Polycomb repressive complex 2 (PRC2) inactivated in T-lymphocyte leukemia cells enhance carcinogenesis effect of NOTCH1 mutations by priming transcriptional activity of Notch1 target genes (Ntziachristos et al., 2012; Simon et al., 2012).

The combination of low-dose DAC (Decitabine) and CS055 (Chidamide) can synergistically induce apoptosis in adult ALL, especially for those with p16 gene deletion through DNA damage (Shi et al., 2017). A retrospective analysis revealed that incorporation of chidamide may improve the prognosis of T-LBL patients with NOTCH1 and RAS/PTEN mutations (Chen F et al., 2020). Chidamide plus chemotherapy group was associated with a significantly better progression-free survival (PFS) than chemotherapy group in refractory or relapsed T-cell acute lymphoblastic lymphoma/leukemia (T-LBL/ALL) (Guan et al., 2019). The combination of decitabine and vorinostat followed by standard re-induction chemotherapy (vincristine, prednisone, doxorubicin, PEG-asparaginase) was tolerable and showed clinical benefit in relapsed patients with ALL (Burke et al., 2014). Pretreatment of allo-HSCT (hematopoietic stem cell transplantation) containing chidamide improves the prognosis of ALL with MRD (Ji et al., 2018). Chidamide also shows good efficacy and tolerability in ETP-ALL and Ph(Philadelphia chromosome)-Like-ALL (Zhou et al., 2018).

The patient was examined for programmed cell death protein 1 (PD-1) expression levels, as well as CD3^+^, CD3^+^CD4^+^, CD3^+^CD8^+^ T cell levels in peripheral blood (Table 1). We found an increase PD-1 expression onCD3+ and CD3^+^CD8^+^ T cells, implicated in regulating T-cell exhaustion (Ngiow et al., 2015; Kim et al., 2018). Although Anti-PD-1 and HDACi are clinically effective in ALL (Bachy and Coiffier, 2013; Burke et al., 2014), there are relatively few studies testing this combination of the two drugs. There is growing evidence that epigenetic alterations can influence immune cell phenotype and function, which trigger the immune response or lead to immune evasion (Chen X et al., 2020). The synergies gained from the HDACi and PD-1 would promote the antigen presentation of tumor-associated antigens (TAAs) on the tumor surface, induce the upregulation of major histocompatibility complex (MHC) molecules and initiate antigen-specific T cell immune responses (Chen X et al., 2020). Targeting epigenetic control factors can restore the function of impaired effector T cell (Schmidl et al., 2018). Sintilimab (anti-PD-1 antibody) plus Chidamide (an oral subtype-selective HDACi) has been shown as a promising treatment with effective antitumor activity and mild toxicity in patients with newly diagnosed Extranodal Natural Killer/T Cell Lymphoma (ENKTL) (Yan et al., 2021). Although an increasing number of clinical trials have tested the efficacy of PD-1/PD-L1 antibodies combined with epigenetic agents based on HDACi treatment of patients with multiple cancer types (Pan and Zheng, 2020), there is no report on the combination therapy of PD-1 and HDACi using in maintenance therapy of T-ALL. We report the first case following chemo-free maintenance therapy with anti-PD-1 antibody plus HDACi for continually 24 months with BM MRD showed continued CR.

## Conclusion and future direction

In summary, we first combined PD-1 and HDACi to treat T-lymphoblastic lymphoma during the maintenance stage to optimize the therapeutic outcomes. The patients received the following maintenance therapy for 2 years: Chidamide (30 mg qW14) + anti-PD-1 (200 mg q3w) and was maintained on remission by bone marrow (BM) evaluation and minimal residual disease (MRD) detected by flow cytometry, which suggest the combination of anti-PD-1 antibody and Chidamide may serve as a promising new Chemo-free therapeutic strategy in the maintenance stage treatment of T-ALL. Future clinical trials were needed to provide the efficiency and safety for this approach.

## Data Availability

The original contributions presented in the study are included in the article/supplementary material, further inquiries can be directed to the corresponding authors.
